# It is time to understand daylight saving time

**DOI:** 10.1093/sleep/zsac309

**Published:** 2023-01-11

**Authors:** José María Martín-Olalla, Jorge Mira

**Affiliations:** Departamento de Física de la Materia Condensada, Facultad de Física, Universidad de Sevilla, Sevilla, Spain; Departamento de Física Aplicada and iMATUS, Universidade de Santiago de Compostela, Santiago de Compostela, Spain

Dear Editor,

*Sleep* recently published a position statement by the Sleep Research Society supporting the abolition of the seasonal clock change, and the adoption of permanent standard time in the United States after “a thorough review of the existing literature” [1]. The beginning section of the statement, devoted to the history of daylight saving time (DST) regulations, shows a key misunderstanding which often occurs within the chronobiological and sleep community. We bring here this letter in an attempt to clarify what is and what is not DST.

Malow [1] attributes to Benjamin Franklin the concept of changing the hours of human activity to “save daylight” and ends saying: “In contrast to what Franklin proposed, where a population wakes earlier to make the best use of daylight, DST changes the clock time. DST shifts daylight into the early evening in exchange for less daylight in the early morning.” There are two things of the utmost importance to note. First, DST does not shift daylight, which is a natural phenomenon alien to human conventions. All else equal, DST changes the phase of human activity: it is advanced in spring and delayed in autumn. As a result of this, the second point to note is: DST regulations exactly achieve what Franklin demanded; the population wakes earlier in summer, and later in winter. In other words: there is no “in contrast” that opposes the current, annoying, DST regulations to Franklin’s prior proposal. Both bring the same main effect: “make the best use of daylight,” meaning people wakes up earlier in summer and later in winter or, only metaphorically, both shift “daylight into the early evening in exchange for less daylight in the early morning.”

There are issues to criticize in connection with the seasonal clock regulations (the stroke of 1 h and the choice of the transition dates, chiefly), but their main effect is not one of those issues because it goes in line with the ancient seasonal practice, and in line with the role of daylight (sunrise) as a synchronizer for the onset of human activity. Therefore, we find interesting that position papers [1] and review papers [2] demand the canceling of the regulation, the adoption of permanent standard time, but, at the same time, they acknowledge the propensity of the population for advancing the activity during the summer (and delaying it back in winter). The thing to note is that since the 20th-century Daylight Saving Time regulations are successfully easing this propensity in modern, Extratropical societies. Else, people would have already delayed their morning times in summer, playing against the regulations [3] or, simply, they would have been deprecated. In this line, we hypothesize that the abolition of the practice will not improve the current scenario in the ranges of latitudes where the contiguous United States locate [4].

Roenneberg et al.’s [2] “potential solution” to daylight saving time consists in the adoption of seasonal start times. In 1810 (only 20 years after Franklin’s death), the Spanish National Assembly already regulated their opening and closing times seasonally (10 am–2 pm from October to April; 9 am–1 pm from May to September) [3], imperfectly mimicing the natural, ancient seasonal adaptation at their circle of latitude, bringing early activity in summer and late activity in winter to representatives. This seasonal behavior has been superseded by the current clock regulations. Both solutions are identical on an individual basis and bring the same hazards. In addition, we must note that during the past 100 years people in the United States, the United Kingdom, and elsewhere, have had every opportunity to offset the clock regulations by moving their start times in opposition (as an example 9 am during the standard time (ST) period and 10 am during the DST period). Nearly nobody behaved like that. Nonetheless, DST is flagged as an “artificial” setting [2, 5].

We do acknowledge that the transition dates should be altered for the benefit of the population. The spring transition date should come after the Equinox so that larger shares of the population do not experience a dark rise time after clocks are changed. An early April transition date, as occurred until 2007 in the United States, would help to mitigate this. Accordingly, if the autumn transition were set to early October, as occurred until 1954, many working population and children would cease to be subjected to the stressing dark hours in the October mornings. See Figure 1 for a graphical sketch of this idea.

**Figure 1.**
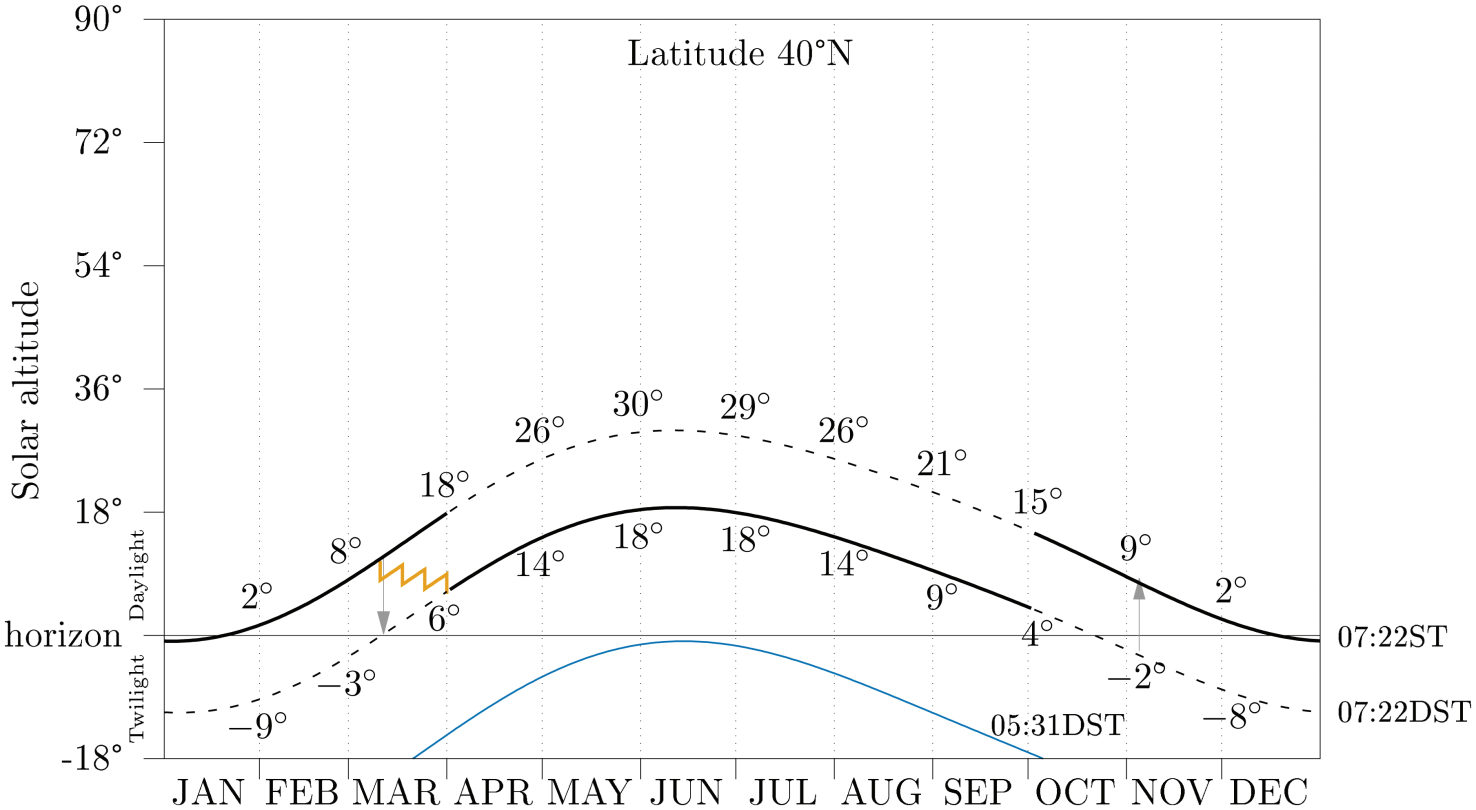
The yearly evolution of the solar altitude at the hour of the winter sunrise (top thick black line, designated 07:22 ST); at 1 h ahead (medium thick black line, designated 07:22 DST or 06:22 ST); and at the summer sunrise (bottom thin blueish line, designated 05:31 DST) for the 40 °N circle of latitude (the latitude of New York and Madrid). The winter sunrise is a synchronizer for the onset of human activity [6, 7]. When DST is set from early April to early October, the onset of the human activity occurs in daylight and delays at most 01 h 51 min from the sunrise. This proposition is noted by solid lines. The vertical arrows annotate the current transition dates in the United States. Numbers inside the graph annotate solar altitude at the beginning of calendar months. The orange zigzag line sketches a four-stroke circadian preadaptation to the spring transition (from the standard clock to the daylight saving clock) achieved by an alarm clock.

The deepest concern of the chronobiological and sleep community lies on the hazards that the stroke of 1 h brings [8–10]. Yet, this is unavoidable after clock time gained significance in modern societies (see the preceding example in Spain): assemblies, schools, companies, and universities can only regulate their start times by whole hours, and not smoothly. Again, DST regulations have provided a simple, effective, socially synchronized mechanism to do so.

Notwithstanding this, individuals can adapt their phase preemptively by altering their alarm clock in the weeks preceding the spring transition. The zigzag line in Figure 1 shows the idea for a four 15-min stroke adaptation. Similar settings for three (20 min) or two (30 min) strokes are possible.

Malow [1] alerts that evening light “extended too close to bedtime can also disrupt sleep patterns.” However, this observation is mainly associated with the shortening of the scotoperiod that the summer brings to Extratropical latitudes. We must note that the winter sunrise time and the summer sunset time are separated by roughly 12 h, irrespective of latitude. If the onset of human activity is determined by the winter sunrise time and clock regulations apply, then the onset of human activity in summer is separated by 11 h from the sunset time, which likely suffices for proper sleep. For those individuals with an onset time earlier than the winter sunrise time, the clock regulations come less handy in summer. Noteworthy, the regulations have also played a role in preventing human activity from starting before the winter sunrise, thus minimizing the size of this group [4].

## Data Availability

The authors confirm that the data supporting this study are available within the manuscript. Sunrise times and solar altitudes in figure 1 were computed with the help of the software ‘xplanet’ by Hari Nair (https://xplanet.sourceforge.net/) to compute the solar declination during the year 2022; and the script ‘Equation of Time’ by Darin C. Koblick (available at MATLAB Central File Exchange https://www.mathworks.com/matlabcentral/fileexchange/32793-equation-of-time) to compute the equation of time.
